# Evaluation of a Mobile Phone App for Patients With Pollen-Related Allergic Rhinitis: Prospective Longitudinal Field Study

**DOI:** 10.2196/15514

**Published:** 2020-04-17

**Authors:** Manuela Glattacker, Martin Boeker, Robin Anger, Frank Reichenbach, Adrian Tassoni, Rainer Bredenkamp, Juergen M Giesler

**Affiliations:** 1 Section of Health Care Research and Rehabilitation Research Medical Center Faculty of Medicine, University of Freiburg Freiburg Germany; 2 Medical Data Science, Institute of Medical Biometry and Medical Statistics Medical Center Faculty of Medicine, University of Freiburg Freiburg Germany; 3 Clinical Trials Unit Medical Center Faculty of Medicine, University of Freiburg Freiburg Germany; 4 Clinical Trials Unit UMG University Medical Center Göttingen Georg-August-University Göttingen Germany

**Keywords:** mobile applications, rhinitis, allergic, seasonal, patient reported outcome measures, prospective studies, longitudinal studies, usability, effectiveness

## Abstract

**Background:**

Mobile health apps have great potential to support the self-management of chronic conditions such as allergic diseases, which constitute significant challenges in health care. However, the health app market is confusing for users, as it is vast, dynamic, and lacks scientific evidence regarding the effectiveness of the apps on offer. To our knowledge, no health app for pollen-related allergic rhinitis has been evaluated.

**Objective:**

The aim of our study was to evaluate the Husteblume mobile phone health app, developed in Germany to facilitate the self-management of pollen-related allergic rhinitis.

**Methods:**

We evaluated usability and changes in quality of life, health literacy, and self-efficacy for managing one’s chronic disease. We conducted 2 online surveys of registered users of the app, 1 before and 1 after the 2017 pollen season, allowing for the analysis of both cross-sectional and longitudinal data in a field setting.

**Results:**

The sample comprised 661 app users at the first measurement point and 143 users at follow-up. The subgroup of study participants at follow-up rated the usability of the app as good or very good. There were no significant changes in patient-reported outcomes such as quality of life, health literacy, and self-efficacy between the 2 measurement points (*P*>.05). However, those reached at follow-up perceived subjective improvements due to the app: 55.9% (80/143) reported being subjectively better informed about their allergy, 27.3% (39/143) noted improved quality of life, 33.6% (48/143) reported subjectively better coping with their allergy, and 28.0% (40/143) felt better prepared for the consultation with their physician. Finally, 90.9% (130/143) users did not identify any adverse effects of the app.

**Conclusions:**

Despite some methodological caveats, the results of the evaluation of the Husteblume app are encouraging for the subgroup using the app in the long term. However, further studies evaluating the effectiveness of the app are needed.

**Trial Registration:**

German Clinical Trials Register DRKS00011897; https://tinyurl.com/yxxrg9av

## Introduction

### Scientific Background

The prevalence of allergic diseases has increased dramatically over the last few decades in many regions of the world [1], and allergic diseases pose a significant challenge in health care [2]. For example, the lifetime prevalence of asthma is 8.6%, and allergic rhinoconjunctivitis, which is a comorbidity in more than 80% of patients with asthma, has a lifetime prevalence of 14.8% [3]. Pollen-related allergic rhinitis is characterized by symptoms such as sneezing, secretion, and conjunctivitis, and is associated with decreased quality of life and performance [3,4]. Effective disease self-management, such as avoiding allergens, and planning medication and everyday life, reduces the burden of pollen-related allergic rhinitis [5]. However, partly due to low adherence to the prescribed medication and a lack of education, allergic rhinitis control is inadequate for many patients [6].

Mobile health (mHealth) apps are a promising way to support the self-management of chronic diseases [7,8]. They have the potential to optimize access to health information and to health interventions in a low-cost way. They can contribute to the empowerment and participation of patients, change health care in a patient-centered, decentralized way, and support health care professionals to treat patients more efficiently [9-11]. Therefore, health apps might increasingly become a “major source of health guidance” ([12] pg 1051) and have the potential to change existing health care delivery pathways [13]. Supporting this, mHealth interventions are growing in popularity worldwide [14]. Over 100,000 mHealth apps are available [10] and are increasingly accepted as a tool to observe and manage health in everyday life [7]. In Germany, a recent population-based survey of more than 4000 participants showed that 61% of participants used a smartphone and, among these, 21% used health apps primarily focusing on smoking cessation, healthy diet, and weight loss [15]. As the mHealth market is one of the fastest-growing areas in health care [16], these numbers will probably continue to increase [17].

However, given the size of and rate of innovation in the health app market, in combination with a lack of objective and valid criteria to assess the quality of health apps [9,16], it can be difficult for end users to choose effective apps. The technical quality of many health apps is problematic, for example, with respect to transparency and data privacy [9,16]. Many apps are developed without the involvement of experts and do not adhere to medical evidence [18]. Further, the lack of evidence regarding the effectiveness of health apps remains a concern. Several reviews have examined the impact of health apps on a specific behavior, such as physical activity [19], adherence to medication [20], or specific diseases such as diabetes [21,22], depression [23], cardiovascular disease [24], chronic renal disease [25], heart failure [26], or chronic obstructive pulmonary disease [27]. Nevertheless, a recent systematic review of 23 systematic reviews assessing the effectiveness of mHealth interventions for different health conditions concluded that the evidence for the efficacy of mHealth interventions is still limited, despite some moderate-quality evidence for improvement across various outcomes [28]. Most of the studies included in the systematic reviews, as well as the reviews themselves, have been criticized for significant methodological limitations [28].

The same is true for health apps for self-management of allergic diseases and asthma. Although the number of apps is growing, there are very few evaluation studies, and the usefulness of these apps is still uncertain [4]. With respect to the effectiveness of apps to facilitate the self-management of patients with asthma, a Cochrane review from 2013 [29] including 2 randomized controlled trials was unable to draw reliable conclusions due to an insufficient number of studies and the considerable degree of heterogeneity between the studies. In 2015, about 200 asthma-related mobile phone apps were available on the iOS and Android platforms [30]. However, a systematic content assessment found that many apps did not include comprehensive information or offer guidance consistent with evidence for asthma self-management. Indeed, 13% of the apps recommended self-care procedures unsupported by evidence [30]. Applying slightly different quality criteria, such as available functions or general quality, a recent review assessing 38 apps found great variation across all of the investigated criteria [31]. Many apps were of low quality, while the major concern was the absence of clinical validation. Finally, a recent systematic review and meta-analysis of 11 studies evaluating the efficacy of mobile technology interventions on clinical outcomes and adherence in individuals with asthma found strong evidence for at least short-term efficacy for asthma management [8]. However, those authors made the criticism that most studies lacked a theoretical basis for their interventions and did not specify the behavior change technique used in the intervention [8].

Very few studies have evaluated the impact of health apps in allergic rhinitis [4]. However, in 2015, a worldwide consortium proposed a plan for the use of mHealth technology in the management of allergic rhinitis (MACVIA-ARIA Sentinel Network [MASK]) [4]. Following this group, the MASK-rhinitis app is the first app to have been tested in a pilot study [32]. To our knowledge, no study has evaluated a health app for pollen-related allergic rhinitis. Such an app would be a potentially effective way to target and supply many people with pollen allergies with pollen information. This would be of great importance, as it could help people to plan medication and everyday life and contribute to a better quality of life [33].

### Objectives

The aim of our study was to evaluate the Husteblume mHealth app for patients with pollen-related allergic rhinitis, with respect to its usability and changes in quality of life, health literacy, and self-efficacy for managing this chronic disease.

## Methods

### The Husteblume Health App

The Husteblume health app was developed by a German health insurance company (Techniker Krankenkasse, Hamburg, Germany). The app is available for download free of charge from the Apple and Google app stores. It aims to support the self-management of its users and provides functions that allow them to (1) register their allergy-related symptoms and their medication in a diary, (2) retrieve prognostic information on the type and amount of pollen expected at the user’s present location, (3) retrieve information about the relationship between the user’s individual symptoms, the pollen load, and the medication the user has been taking and then graphically display this information for a period of time (week or month), (4) access information about available treatments for a specific type of pollen allergy and its symptom burden, (5) access a dictionary providing information on allergens and cross-allergies, and (6) perform a self-test to assess their allergic rhinitis.

As is apparent from these features, the app uses behavior change techniques [8], most notably *information about antecedents* of symptoms, *information about consequences* of behaviors*,* and *self-monitoring.* It thus allows deliberate planning of behaviors aimed at avoiding or otherwise managing situations that tend to increase rhinitis symptoms. The app was developed on the basis of current medical guidelines and provides information on its functionality and accountability (eg, the security and privacy of user data and the timeliness of the information that the app provides).

### Design, Recruitment, and Ethics

We conducted an online survey during the 2017 pollen season using a design with 2 measurement points that combined a cross-sectional and a longitudinal approach in a field setting.

To be eligible, participants had to be registered users of the app, aged 18 years or older, and allergic to birch or grass pollen, or both, as these most frequently elicit allergic rhinitis. The first measurement was taken before the allergen season (T0) started and the second one, after it had ended (T1). Since the birch and grass pollen seasons cover the period from late March to mid-May and from mid-May to July, respectively, we set the first measurement point to April 1 and the second to August 31. We chose this time interval to ensure that app users would have a sufficiently broad basis of experience to rate the usability of the app and possible changes in patient-reported outcomes.

Potential participants were contacted via 3 routes. During the release of an update of the app by the provider, users were notified of the study via a teaser within the app and asked to participate. Potential users were notified of the study through a push message when browsing the provider’s website that generally aims to provide insurees with information on insurance benefits. In addition, the app provider referred potential participants to the study via various print materials.

If potential users agreed to participate from within the app, they immediately were linked to the survey website at our institution. There, they were presented with detailed information about the study. If consenting to participate, users had to indicate this by ticking 3 consent boxes stating they were at least 18 years old, agreed with the data protection statement, and agreed to participate in the study. Thereafter, they were immediately referred to the survey. Multimedia Appendix 1 shows the challenges of programming the survey.

The study received approval from the Ethics Committee of the University of Freiburg (reference number 33/17) and was registered with the German Clinical Trials Register (DRKS00011897).

### Measures

We measured all variables using either self-constructed items or validated questionnaires. For this paper, 2 of the authors (JMG, MG) translated the survey questions and responses used from German to English.

To measure participants’ *access to the app* and their *previous use* of health apps, we used 4 items at T0. These covered (1) access routes, (2) previous use of the app, (3) if used, frequency and duration of previous use of the app, and (4) use of other health apps.

*Sociodemographic variables* (assessed at T0) included age, sex, nationality, marital status, whether living with a partner, education, and occupational status.

*Allergy- and treatment-related variables* (measured at T0) covered physician-certified allergy diagnosis, type of allergy, allergen(s) with hyperreactivity, time since allergy onset, degree of impairment during pollen season, use of medication, comorbidities (eg, asthma or sinusitis), and smoking status.

We measured *usability* at T1 using 13 items that were based on 2 established instruments: the System Usability Scale (SUS) [34] and the Modular Evaluation of Components of User Experience (meCUE) [35]. Further, the qualitative work of Grindrod and colleagues [36] provided useful information on dimensions of mobile app user experiences. Therefore, we used items addressing the dimensions of personal usefulness, simplicity, accessibility, functionality, and design of the app. However, since we found the wording of the German translations of both the SUS [37] and the meCUE [35] not entirely satisfactory, we decided to write new items addressing content that was in part covered by the SUS (4 items) or the meCUE (5 items). Finally, we added 1 item asking for a summary evaluation of the app. All the usability items were answered on 5-point scales ranging from “not at all true” to “completely true” with a middle category of “partly true, partly not true”.

We measured *user behavior during the pollen season* at T1 using 2 self-constructed items. These required participants to rate their average frequency of app usage per week during the pollen season and to report whether they had used the app in relation to 3 levels of symptom burden (low, medium, and high, multiple responses possible).

We measured *perceived effects* of using the app *on self-management and illness behavior* by self-constructed items at T1 that focused on changes participants might have perceived as resulting from using the app during the pollen season. These items covered the following aspects: (1) participants’ knowledge about the allergy, (2) frequency of health service consultations due to the disease, (3) form of preparation for health service consultations, (4) experience of negative effects due to the app, (5) adherence to physicians’ advice, (6) management of the allergy, (7) perceived improvements to their condition, and (8) perceived improvements to their quality of life in general. Responses to these items were measured on 5-point scales ranging either from “not at all true” to “completely true” or from “deteriorated” to “improved”.

We measured *health-related quality of life* at T0 and T1 using the Quality of Life in Allergic Rhinitis (FL-Heu) questionnaire [38]. The FL-Heu consists of 32 items combined into 7 scales measuring quality of life in terms of impairment in various domains and 1 generic item addressing the respondent’s current health in general. The scales cover the domains of sleep, eyes, nose, general symptoms, social relationships, being affected by the disease, and emotional impairment. Items are answered on a 7-point scale. Higher item and scale scores represent greater impairment and thus lower quality of life. Scale consistencies (Cronbach alphas) have been reported to range from .74 to .90. The instrument has been found to be sensitive to change [38,39].

We measured *health literacy* at both time points with the Health Education Literacy of Patients With Chronic Musculoskeletal Diseases (HELP) [40] questionnaire. HELP consists of 18 items combined into 3 scales measuring comprehension of medical information, applying medical information, and communicative competence in patient-provider interactions. Scale internal consistencies (Cronbach alpha) range from .88 to .95. The scales are compatible with a Rasch model and preliminary evidence of their validity is available [40]. Higher scale scores represent higher levels of health literacy.

We measured *self-efficacy for managing one’s chronic condition* at T0 and T1 with a scale by Lorig [41,42]. Its 6 items ask how confident one feels that one can do various things without interference from one’s chronic condition. Items are answered on a 10-point scale ranging from “not at all confident” to “totally confident.” An individual’s scale score is represented by the mean of their item responses. The Cronbach alphas reported for this scale exceed .90 [41,42]. Higher scores indicate higher self-efficacy.

In total, the survey included 92 items at T0 and 85 items at T1. At each time point, questions were presented in a linear order across a total of 17 pages at T0 and 16 pages at T1. In some instances, we used adaptive questioning (ie, in regard to nonemployment).

### Data Analysis

Data analysis included all participants who had provided data at either the first or the first *and* second measurement points. First, we computed descriptive statistics for sociodemographic, medical, and app access parameters measured at T0. We also determined descriptive statistics for user behaviors and perceived effects and changes in health and illness behaviors measured at T1. Missing data were not imputed. When computing scale scores, we handled missing data in compliance with the recommendations of the authors of the respective questionnaire. To estimate whether completers of the survey differed from dropouts in respect to the characteristics measured at T0, we computed chi-square analyses and *t* tests for independent groups. To determine changes from T0 to T1, we performed paired *t* tests (2-tailed) for quality of life, health literacy, and self-efficacy for managing one’s chronic condition. We set type I error probability to *P*=.05 throughout. All computations were performed with the statistical software IBM SPSS version 25 (IBM Corporation).

## Results

### Participant Flow, Sample Characteristics, and Dropout Comparisons

**Figure 1:**
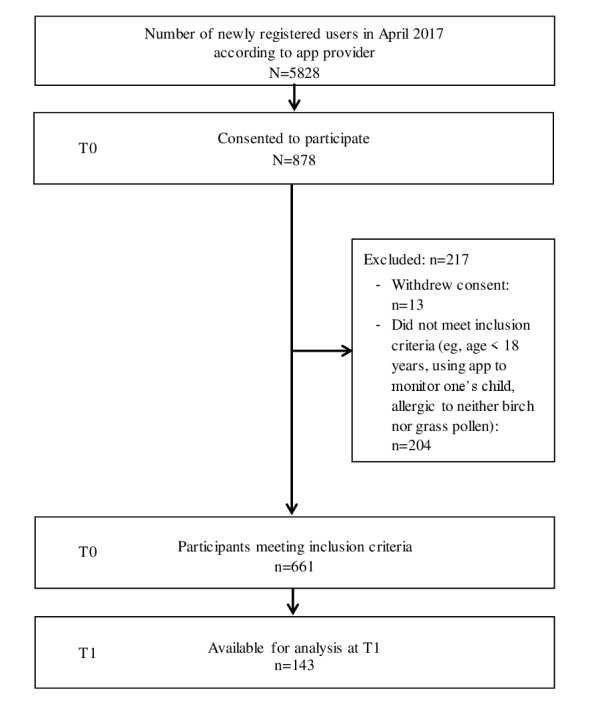
Participant flow from first (T0) to second measurement point (T1).

Figure 1 presents the participant flow from the first (T0) to the second point of measurement (T1). At T0, 5828 persons had registered as users of the app. Of these, 878 (15.1%) consented to participate; 13 withdrew their consent and thus were excluded from the study. In addition, 204 persons did not meet the inclusion criteria and were excluded. Thus, at T0 data from 661 individuals were available for analysis.

At the second point of measurement, 143 persons completed the study (21.6% of those participating at T0 and 2.45% of those who initially had registered for the app). It should be noted, however, that the total number of active users of the app decreased drastically from registration to T1, that is, from 5828 in April to 191 in August 2017. From this perspective, the proportion of participants at T1 was 74.9% of those who were still actively using the app at that time.

It took participants a mean of 11 (SD 6.65) minutes at T0 and 10 (SD 6.01) minutes at T1 to complete the survey. At T0, 91.8% (607/661) of those starting the survey completed it; at T1 this proportion was 88.8% (127/143).

Participants were a mean of 39 years old, and 58.6% (387/660) were female (see Table 1 for more information on sociodemographic and medical characteristics). A total of 73.2% (484/661) rated their impairment during the pollen season as strong or very strong (results not displayed in Table 1). Of the sample, 24.7% (163/661) had not used the app before, and 71.9% (358/498) of those who had previously used the app indicated they had done so for 3 to 4 or for 5 or more days per week. A total of 53.7% (355/661) reported not currently using any other health apps.

Comparisons between dropouts (n=518) and completers (n=143) of the study indicated that the 2 groups were comparable with regard to most of the variables. The only significant difference was that completers were older on average than dropouts (mean age 42.0, SD 21.0 vs mean 38.6, SD 12.7 years, respectively; *t*_659_=2.82; *P*<.01).

**Table 1:** Sociodemographic and medical sample characteristics (N=661, unless otherwise indicated).

Characteristics			Values
Age (years), mean (SD)			39.4 (12.6)
**Sex (n=660), n (%)**			
	Male	273 (41.4)	
	Female	387 (58.6)	
**Marital status, n (%)**			
	Single	290 (43.9)	
	Married	327 (49.5)	
	Divorced or separated	40 (6.1)	
	Widowed	4 (0.6)	
**Years of education, n (%)**			
	9	23 (3.5)	
	10	144 (21.8)	
	11	14 (2.1)	
	12	100 (15.1)	
	13	371 (56.1)	
	0	2 (0.3)	
	Other	7 (1.1)	
**Employed, n (%)**			
	Yes	579 (87.6)	
	No	82 (12.4)	
**Allergy diagnosis by physician, n (%)**			
	Yes	648 (98.0)	
	No	13 (2.0)	
**Allergen, n (%)**			
	Birch pollen (only)	165 (25.0)	
	Birch and grass pollen	398 (60.2)	
	Grass pollen (only)	98 (14.8)	
**Duration of allergy (years), n (%)**			
	<1	5 (0.8)	
	1-4	88 (13.3)	
	5-10	131 (19.8)	
	>10	437 (66.1)	
**Use of medication against allergic rhinitis (n=657), n (%)**			
	Yes	589 (89.6)	
	No	68 (10.4)	

### App Usage During Pollen Season and Usability

Of those participating in the study at T1, 73.4% (105/143) reported having used the app during the pollen season for 3 to 4, or for 5 or more days per week. A total of 87.4% (125/143) rated the app as easy to use and 1.4% (2/143) rated it as too complicated or requiring too much prior knowledge (Table 2). Access to the app was rated positively or very positively by 93.0% (133/143) of the participants, and a similar proportion provided an overall positive evaluation. The functionality of the app, its design, and the personal benefit of using it also received positive ratings; for example, 84.6.% (121/146) of participants at T1 intended to use the app in the future. However, there was more variation between these items and lower proportions of respondents agreed with them.

**Table 2:** Descriptive statistics of app usability items (137≤n≤143; in descending order of means).

Dimensions and items (abbreviated)			Answer scores						
			Mean (SD)		(mostly) not true, n (%)^a^		partly true, partly not true, n (%)^a^		(mostly) true, n (%)^a^
**Simplicity**									
	Easy to see how to operate the app^b^	4.42 (0.68)		2 (1.4)		9 (6.3)		126 (88.1)	
	App easy to handle^b^	4.33 (0.73)		2 (1.4)		16 (11.2)		125 (87.4)	
	App too complicated^b^	1.67 (0.86)		126 (88.1)		10 (7.0)		7 (4.9)	
	App requires too much prior knowledge to be operated effectively^b^	1.53 (0.73)		126 (88.1)		7 (4.9)		4 (2.8)	
**Functionality**									
	Results reporting clearly arranged^b^	3.87 (0.83)		9 (6.3)		33 (23.1)		101 (70.6)	
	Functions well integrated^b^	3.80 (0.75)		6 (4.2)		36 (25.2)		95 (66.4)	
**Personal benefit**									
	Will use app in the future^b^	4.15 (0.78)		22 (15.4)		16 (11.2)		121 (84.6)	
	App is very useful^b^	4.01 (0.79)		4 (2.8)		31 (21.7)		108 (75.5)	
	Functions appropriate for my goals^b^	3.74 (0.93)		14 (9.8)		36 (25.2)		87 (60.8)	
	App provides much useful information^b^	3.63 (0.85)		12 (8.4)		48 (33.6)		77 (53.8)	
**Design**									
	App design attractive^b^	3.89 (0.75)		5 (3.5)		34 (23.8)		104 (72.7)	
**Access**									
	App easy to download and install^b^	4.79 (0.57)		2 (1.4)		2 (1.4)		133 (93.0)	
**Overall evaluation^a^**									
	All in all, app is running well^b^	4.48 (0.63)		2 (1.4)		4 (2.8)		131 (91.6)	

^a^Percentages were computed based on all 143 respondents at T1. This includes respondents who had missing data for individual variables. Thus, row percentages do not always add up to 100%.

^b^Response categories and scores: “not true at all” (1), “mostly not true” (2), “partly true, partly not true” (3), “mostly true” (4), “completely true” (5).

### Perceived Changes

A total of 2.1% (3/143) of participants reported negative effects of the app (Table 3). By contrast, 55.9% (80/143) felt better informed about their rhinitis, and between 20.3% (29/143) and 33.6% (48/143) indicated that they felt supported with respect to adherence to medication, preparing for medical visits, or coping. The vast majority at least partially agreed that using the app had led to improvements in their quality of life or their allergic rhinitis.

**Table 3:** Descriptive statistics of items assessing perceived changes in information, coping, medical visits, adherence, quality of life, and negative effects as a consequence of app use (n=133).

Using the app...	Answer scores			
	Mean (SD)	(mostly) not true, n (%)^a^	partly true, partly not true, n (%)^a^	(mostly) true, n (%)^a^
makes me feel better informed about my allergic rhinitis^b^	3.59 (1.00)	22 (15.4)	31 (21.7)	80 (55.9)
helps me cope better^b^	3.11 (1.04)	36 (25.2)	49 (34.3)	48 (33.6)
helps me prepare better for my medical visits^b^	2.88 (1.11)	46 (32.2)	47 (32.9)	40 (28.0)
helps me adhere to my doctor’s recommendations^b^	2.62 (1.09)	64 (44.8)	40 (28.0)	29 (20.3)
improved my quality of life^c^	3.32 (0.53)	0 (0.0)	94 (65.7)	39 (27.3)
improved my allergic rhinitis^c^	3.10 (0.30)	0 (0.0)	120 (83.9)	13 (9.1)
has negative effects on me, too^b^	1.36 (0.62)	140 (90.9)	1 (0.7)	2 (1.4)

^a^Percentages were computed based on all 143 respondents at T1, including those with missing data for individual variables. Thus, row percentages do not always add up to 100%.

^b^Response categories and scores: “not true at all” (1), “mostly not true” (2), “partly true, partly not true” (3), “mostly true” (4), “completely true” (5).

^c^Response categories and scores: “worsened” (1), “worsened somewhat” (2), “neither worsened nor improved” (3), “improved somewhat” (4), “improved” (5).

### Changes in Quality of Life, Health Literacy, and Self-Efficacy from T0 to T1

As Table 4 shows, we detected almost no significant changes between the 2 measurement points for quality of life variables, health literacy, or self-efficacy for managing one’s chronic condition; the only exception was less impairment of quality of life by nasal symptoms.

**Table 4:** Changes in rhinitis-related quality of life, health literacy, and self-efficacy of coping with chronic disease across the observation period (116≤n≤127).

Subscale and attribute		T0 score, mean (SD)	T1 score, mean (SD)	*r*	*t*	*df*	*P* value
**Impairment of quality of life**							
	Sleep^a^	34.48 (19.23)	34.48 (22.68)	.538	0.00	120	>.99
	Eyes^a^	39.38 (21.57)	35.85 (20.94)	.490	1.79	118	.08
	Nose^a^	62.18 (21.11)	57.45 (24.26)	.483	2.23	119	.03
	General symptoms^a^	41.32 (18.11)	40.70 (18.14)	.625	0.43	118	.67
	Social relationships^a^	39.05 (19.77)	37.75 (20.93)	.681	0.88	118	.38
	Impairment through disease^a^	54.35 (20.66)	53.81 (20.19)	.530	0.29	115	.77
	Emotional impairment^a^	36.68 (17.12)	35.19 (18.21)	.642	1.08	117	.28
	General health^b^	3.58 (1.32)	3.33 (1.35)	.281	1.67	117	.10
**Health literacy^c^**							
	Understand medical information	82.28 (16.26)	80.54 (17.76)	.722	1.54	126	.13
	Apply medical information	85.28 (13.92)	83.19 (15.50)	.551	1.68	126	.10
	Talk to clinicians	80.88 (19.33)	78.74 (21.03)	.686	1.50	126	.14
**Self-efficacy^d^**							
	Managing chronic disease	7.29 (1.90)	7.36 (1.75)	.636	–0.48	125	.63

^a^Scale scores range from 0 to 100. Higher scores indicate greater impairment.

^b^Scores range from 0 to 7. Higher scores indicate greater impairment.

^c^Scores range from 0 to 100. Higher scores indicate higher health literacy.

^d^Scores range from 1 to 10. Higher scores indicate higher self-efficacy beliefs.

## Discussion

### Principal Findings

We are not aware of any studies evaluating a health app in the context of pollen-related allergic rhinitis, and there are few German health app studies in the literature [43]. Therefore, the aim of our study was to evaluate the Husteblume mobile app among patients with pollen-related allergic rhinitis with respect to its usability and changes in patient-reported outcomes.

The Husteblume app meets many of the quality criteria that have been suggested to determine the quality of health apps [12,44]. It was developed on the basis of current medical guidelines and provides functionality, credibility, and accountability information. Pollen-specific quality criteria such as providing comprehensive information on pollination, guiding management of the pollen allergy, allowing the documentation of symptoms, and informing the user about the developer of the app [33] are implemented. The app uses several behavior change techniques [8] with a focus on self-monitoring, one of the most common behavior change techniques applied in apps across a broad range of health issues [11].

The major results of our study showed that the usability of the app was largely rated positively by its users. While we observed few significant changes in patient-reported outcomes over time, participants indicated subjectively perceived changes of varying degree in relation to being better informed about their condition, to better coping with it, or to their quality of life.

Usability, which is a critical factor for the continuous application and the effectiveness of health apps [43] and is therefore part of several taxonomies for assessing health apps [12,44], was rated as good or very good in the subgroup of study participants who were followed up. This result might also be responsible for their comparatively high level of adherence to the app. Of those we followed up, 51.0% (73/143) used the app for more than 6 months, and 84.6% (121/143) of users who were reached at the second measurement time point were motivated to use the app in the future. However, in line with studies showing that many stop using a health app shortly after downloading it [45], a high number of participants dropped out of the study. The number of active users of the app decreased from 5828 in April to 191 in August 2017. This resulted in only 2.45% (143/5828) of those who had registered for the app still being involved in its evaluation at follow-up. While the proportion of ongoing users who were followed up was relatively high (143/191, 74.9%), findings regarding the usability of the app were gained in a relatively small subgroup of long-term active users. We do not know whether those who stopped using the app were discouraged by a perceived lack of usability or whether there were other reasons for dropping out from active use, such as the timing of the T1 assessment. The T1 assessment took place well after the end of the pollen season, and these people with allergy might have had little reason to continue using the app after the pollen season had finished. Thus, in future studies it would be useful to analyze users’ reasons for ceasing to use the app.

With respect to patient-reported outcomes, such as quality of life, health literacy, and self-efficacy, there were no significant changes between the 2 measurement points. Nevertheless, those participants who were followed up perceived subjective improvements due to the app; half felt better informed about the allergy, one-quarter reported improved quality of life, and one-third reported subjectively better allergy self-management and being better prepared for the consultation with their physician. Finally, most users could not identify any adverse effects of the app. However, the high dropout rate should be kept in mind when interpreting these findings.

### Strengths and Limitations

The results of our study must be interpreted in the light of 3 major limitations. The first limitation is that selection bias may reduce the generalizability of the results. Initially, only 15.07% (878/5828) of all registered Husteblume app users could be reached as study participants, so the results cannot be generalized to all users. By follow-up, the number of active users of the app had decreased drastically since registration, and the participation rate of 21.6% (143/661) of the initially included study participants further limits the generalizability of the results. Having said that, with the exception of younger age, there were no significant differences between dropouts and those who were followed up. Moreover, we were able to reach 74.9% (143/191) of the active users at the time of the follow-up assessment. Nonetheless, our results predominantly represent the situation in a relatively small subgroup of long-term active users.

Compared with the MASK sample of more than 2500 users from 20 countries [32], our sample comprised a comparatively high proportion of women (59% versus 43% in the MASK study), and slightly older users (mean age 39 years versus 33 years in the MASK study). Our sample was characterized by high educational status and long-term patients who had allergy for over 10 years. While these characteristics may have affected the results of the evaluation to some extent, the direction of the influence is not clear. On the one hand, the literature shows that selection bias is a problem for many studies using information technology tools [32], as well as for the technology tools themselves. On the other hand, the role of patient characteristics such as age, sex, or disease severity in the use and effectiveness of health apps is still unclear [46]. Therefore, further studies focusing on underrepresented patient groups are needed.

The second major limitation relates to design aspects of our study. We applied a single-arm, noncontrolled study design. Similar study designs are common in evaluations of, for example, asthma-related health apps [47], and there are several challenges associated with designing evaluation studies for health apps [9]. Barriers to evaluation can be seen in the mismatch between the rapid pace of mHealth innovation and rather rigid research designs; in the difficulty of applying characteristics of gold-standard research designs (eg, randomized controlled trial) such as blinding; in the selection of the appropriate app-related outcome variables such as patient autonomy, transparency, or satisfaction with information; and in the lack of psychometrically sound measures of many of these outcome domains [9,48]. While trials of higher methodological quality are needed [11], the appropriate research standard in this area is still being debated [49]. Taken together, external or ecological validity needs to be maximized without reducing the study’s internal validity [50].

Concerning the design of our study, that we refrained from reporting power analyses due to practical aspects of the study can also be criticized. Our potential sample was a priori limited to those who registered for the app during the 2017 pollen season. However, a power analysis for paired *t* tests showed that a sample of 115 participants would be required to detect small effects (of 0.24 with an alpha of .05 and 1–beta of .80). Therefore, the study was adequately powered.

Furthermore, while we used validated tools for the assessment of changes in quality of life, health literacy, self-efficacy, and—in part—usability, we also used self-constructed single items, for example, to assess perceived changes. The development of these items was based on our experience with similar studies and on clinical expertise; however, we did not pilot test or validate the items beforehand.

A challenge in the context of the evaluation of a pollen-related health app is to select the optimal measurement time points, which ensure that disease burden or quality of life (which were outcomes in our study) do not decrease simply due to seasonal differences in pollination. With our study design, this cannot be completely excluded. Applying the selected measurement points, we attempted to cover the start and the end of the pollen season for both birch and grass pollen allergies, ensuring that app users had a sufficiently broad basis of experience to rate the usability of the app and perceived changes in health-related outcomes. However, the third major limitation of this study is that the timing of the T1 assessment may have been too late. The vast majority of users had stopped using the app by that time, and in addition to the possibility that they didn’t find the app to be useful, it is also plausible that they stopped using the app because the pollen season was over and they no longer needed the app.

The strengths of our study include the relatively large sample, the assessment of the majority of active users at follow-up, and the combination of a cross-sectional and longitudinal study design focusing on different outcome measures, such as usability and patient-reported outcomes.

Bearing in mind that health apps with even a small positive effect on health might still be a valuable intervention if the population-level reach is high [11], we conclude that the results of the evaluation of the Husteblume app are encouraging. However, further studies addressing the abovementioned limitations are needed.

### Conclusion

Despite the obvious potential of health apps, high-quality apps are still rare. Evidence is still lacking for their usability, integration into treatment processes, and effectiveness.

To our knowledge, this is the first study to evaluate a health app for pollen-related allergic rhinitis. Despite limitations due to methodological problems, the study showed that the subgroup of study participants at follow-up rated the usability of the Husteblume app as good, and that these users perceived many subjective improvements due to the app. Therefore, we conclude that the results of the evaluation of the Husteblume app are encouraging, but that further studies evaluating the effectiveness of the app are needed.

## Appendix

Multimedia Appendix 1 Challenges associated with programming the survey.

## Abbreviations

FL-Heu: Quality of Life in Allergic Rhinitis
HELP: Health Education Literacy of Patients With Chronic Musculoskeletal Diseases
MASK: MACVIA-ARIA Sentinel Network
meCUE: Modular Evaluation of Components of User Experience
mHealth: mobile health
SUS: System Usability Scale
