# Supplementary material for: Evaluation of a Mobile Phone App for Patients With Pollen-Related Allergic Rhinitis: Prospective Longitudinal Field Study
Source: JMIR Mhealth Uhealth. 2020 Apr 17;8(4):e15514. doi: 10.2196/15514 (PMC7195669; doi:10.2196/15514)
Supplement: Multimedia Appendix 1 [file mhealth_v8i4e15514_app1.docx]

Table M1. Challenges associated with programming the survey

| Programming the survey faced three major challenges:   - Ensuring the anonymity of participants, while also: - Matching their responses from the two measurement points, and - Ensuring that survey data collected by the research team and data available to the app provider from the user registration process could not be linked.   To achieve this, the following procedure was implemented. After a potential participant had agreed to participate from within the teaser, a random number was generated that served as a temporary pseudonymous identifier (ID) valid for 1.5 hours and was stored by both the app provider and the survey site. If the potential participant subsequently consented to participate, the app provider was notified accordingly. The app provider then generated and stored a (again pseudonymous) “technical ID” and forwarded it to the survey site, where it replaced the earlier random number. Based on this technical ID the app provider could alert participants to the second measurement point and refer them to the second survey. It was therefore also possible for the survey site to match a participant’s responses from each survey. After data collection had been completed the technical ID was removed from the data base at the survey site. From this point, available data were completely anonymous. |
| --- |
